# Estimating enrichment of repetitive elements from high-throughput sequence data

**DOI:** 10.1186/gb-2010-11-6-r69

**Published:** 2010-06-28

**Authors:** Daniel S Day, Lovelace J Luquette, Peter J Park, Peter V Kharchenko

**Affiliations:** 1 Harvard-MIT Health Sciences and Technology, 77 Massachusetts Avenue, Cambridge, MA 02139, USA; 2 Center for Biomedical Informatics, Harvard Medical School, 275 Longwood Avenue, Boston, MA 02115, USA; 3 Informatics Program at Children's Hospital, 300 Longwood Avenue, Boston, MA 02115, USA

## Abstract

We describe computational methods for analysis of repetitive elements from short-read sequencing data, and apply them to study histone modifications associated with the repetitive elements in human and mouse cells. Our results demonstrate that while accurate enrichment estimates can be obtained for individual repeat types and small sets of repeat instances, there are distinct combinatorial patterns of chromatin marks associated with major annotated repeat families, including H3K27me3/H3K9me3 differences among the endogenous retroviral element classes.

## Background

Recent progress in high-throughput sequencing platforms has led to widespread utilization of short-read sequencing measurements for functional characterization of genomes. Application of high-throughput sequencing to chromatin immunoprecipitation (ChIP-seq) profiling has been particularly successful, owing to its high signal-to-noise ratio, a higher genome coverage, and better spatial accuracy compared to the established microarray alternative [1]. The ChIP-seq technique has already been used to assess binding patterns of numerous transcription factors, histone modifications, and variants across large mammalian genomes [2-4]. It is also being employed by large-scale functional profiling efforts, such as the ENCODE (Encyclopedia of DNA Elements) project [5,6].

The processing of the ChIP-seq data involves alignment of the reads to the genome, followed by evaluation of the read density patterns to identify regions of statistically significant enrichment that indicate presence of the queried epitope. A number of computational methods have been proposed for such analysis [7-9]. However, these methods typically utilize only the reads for which a unique alignment to the reference can be obtained. Positions corresponding to non-unique (repetitive) sequences are masked, as specific binding at these loci cannot be assessed [10].

The functional properties of the repetitive sequences, however, are of significant biological interest. The repetitive elements comprise significant fractions of eukaryotic genomes, including more than half of the human genome. These elements play important roles in structural organization of the chromosome, gene regulation, and the evolutionary dynamics of the genome [11-14]. Recent studies have shown that some repetitive elements contain evolutionarily conserved transcription factor binding sites and most likely participate in the regulation of specific genes [15,16]. However, activation of many repetitive elements, such as endogenous retroviruses (ERVs), can be deleterious to gene regulation and has been linked to a number of diseases [17,18]. To guard against harmful effects of insertions and rearrangements associated with the presence of transposable elements, the organisms have evolved a variety of defense strategies, including epigenetic mechanisms mediated by RNA interference, DNA methylation, and histone modifications [19,20]. Assessment of the epigenetic states associated with the repetitive elements is therefore of particular interest.

Here, we describe computational methods for enrichment analysis of the repetitive elements, taking advantage of the increased coverage of those elements made possible by high-throughput sequencing. For the microarray platforms, such analysis posed a number of serious difficulties, since the presence of probes with high degrees of sequence homology and large variations in copy numbers led to increased signal intensity range and cross-hybridization. Earlier studies have therefore relied on directed ChIP using primers designed to amplify canonical repeat sequences - prototypical sequences, usually representing a consensus sequence of a known repeat type [21-23].

The developed methods include an improved approach for estimating read enrichment associated with annotated repeat types, and a novel phylogenetic approach for general analysis of enrichment in sequences with a high degree of sequence identity. While sequencing data have been used previously to estimate average enrichment for major repeat families [4,24], such analysis was based only on the canonical repeat sequences. The genome-wide coverage of sequencing data provides information about repetitive sequences beyond that captured by the canonical sequences, and our method, which incorporates sequence variations on those canonical sequences, results in a more than ten-fold increase in the number of sequence reads utilized for repeat sequence analysis. We note that the current analysis is focused on known repetitive elements, and does not attempt to identify novel repetitive sequences.

We apply these methods to analyze histone modifications in human and mouse cells. Our results illustrate that informative enrichment estimates can be obtained for specific repeat types and, in many cases, for small groups of individual repeat instances. We find that sequences associated with many known repeat families exhibit distinct combinatorial patterns of chromatin marks. While we focus on ChIP-seq data, this analysis framework can be extended to analysis of copy number variation and other applications.

## Results

### Incorporating ambiguously and uniquely mapped reads

**Figure 1:**
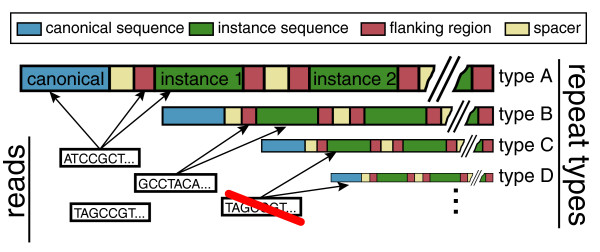
**Aggregating reads for repeat type enrichment estimation**. To increase the accuracy, the enrichment estimate combines reads mapping to the canonical repeat sequence and reads mapping to the body or boundaries of the repeat instances incorporated into the genome assembly. The calculation utilizes reads that align to multiple positions in the genome, if all such positions belong to the instances of the same repeat type. Reads aligning to more than one repeat type are excluded.

Earlier studies have examined enrichment estimates for a given repeat type based on the reads mapping to the canonical sequence of that repeat [4,24]. Since the assembled genome incorporates instances of most annotated repeat types, we can also take into account the reads that map into the repeat instance body or boundary regions (Figure 1). These unique alignments are possible because of the mutations that have accumulated within individual repeat sequences, and the unique sequences of flanking repeats. In the other cases, the mapping remains ambiguous. In estimating the average enrichment for a particular repeat type, however, a read with multiple potential alignments can be taken into account if all of the regions to which it aligns belong to the instances of the same repeat type (Figure 1; see Materials and methods). It is important to note that the methods described in this section estimate an average enrichment for a set of homologous repeat sequences, and do not provide information about the variability of enrichment within the set. The resulting estimates may, for instance, be driven by a small subset of sequences belonging to a given repeat type. Because the number of copies for a given repeat type is typically unknown and can vary between cells, the enrichment calculations rely on input sequencing to normalize the read counts associated with each repeat (see Materials and methods).

To compare different methods for estimating repeat enrichment, we have employed data on several histone methylation marks in mouse embryonic stem (mES) cells measured by Mikkelsen *et al. *[4]. We first compared the enrichment estimates based on the reads mapping to canonical repeat sequences with the estimates based on the reads mapping to repeat instances and associated boundary regions. The incorporation of the instance-mapped reads leads to the expected improvement in the ability to resolve enrichment estimates (Table 1). For instance, the alignment of mES cell histone H3 ChIP reads to the canonical repeat sequences leaves 57% of repeat types without any uniquely-associated reads (70% with less than 10 reads). Combining reads mapping to both canonical and repeat instance sequences increases the number of utilized reads approximately 10-fold, leaving only 4% of repeat types without any reads (20% with less than 10 reads). Consequently, predictions on combined read counts result in several fold increase in the number of repeat types for which a statistically significant level of H3K9me3 enrichment or depletion can be established.

**Table 1:** Combining reads mapping to canonical and instance sequences improves ability to resolve enrichment levels

	Canonical	Instance	Combined
Total number of input reads utilized	150,377	1,270,497	1,498,796
Fraction of repeat types with 0 input reads	0.57	0.05	0.04
Fraction of repeat types with less than 10 input reads	0.71	0.20	0.20
Fraction of repeat types with significant enrichment or depletion	0.20	0.69	0.69
Fraction of repeat types with CI less than 1	0.14	0.57	0.57
Median size of the CI	14.67	0.63	0.58
Mean size of the CI	9.25	1.87	1.77

Using mES H3K9me3 data, the table shows that incorporating reads mapping to the repeat instances allows enrichment estimates to be provided for a large number of repeat types for which canonical sequence alone is not sufficient. CI refers to a 95% confidence interval.

To assess the agreement between canonical and instance-based enrichment estimates, we have selected repeat types for which statistically significant enrichment or depletion can be established based on the canonical sequences (using a 0.05 *P*-value threshold) and determined how often the confidence intervals of canonical and instance-based enrichment estimates overlap (Table 2; see Tables S1 and S2 and Figure S1 in Additional file 1 for other significance thresholds). While the agreement varies slightly for different histone marks, canonical and instance estimates are consistent in approximately 70% of the cases. We will therefore evaluate enrichment using combined canonical and instance sequences, since this allows utilization of a significantly greater portion of available reads and, consequently, provides more informative estimates.

**Table 2:** Agreement between enrichment estimates based on reads mapping to canonical and instance sequences

	H3K27me3	H3K36me3	H3K4me3	H3K9me3	H4K20me3	RNA Pol II
Fraction of repeat types with intersecting canonical and instance-based CIs	0.84	0.93	0.84	0.93	0.92	0.63
Average ratio of canonical and instance-based CI sizes	25.6	24.5	25.9	22.5	22.1	20.0

Using mES data for different chromatin marks, the table shows the fraction of repeat types for which estimates based on canonical and instance read counts were statistically consistent. Only repeat types for which the canonical confidence interval (CI) did not include 0 (that is, significant enrichment was detected) were considered in the analysis. The canonical and instance-based estimates are typically consistent for 70% of the repeat types; however, instance-based estimates provide, on average, much smaller confidence intervals.

The read mapping procedure (Figure 1) guarantees to select reads that can be uniquely associated with repeat sequences belonging to a single repeat type. However, as detection of repeat instances in the genome assemblies is a challenging problem, there are typically sequences within the genome that have a high degree of sequence identity with a given repeat type, yet are not included among the list of annotated instances of that repeat type. If such sequences are not representative of a given repeat type in terms of their biological characteristics, their inclusion could bias downstream analysis. On the other hand, exclusion of all reads that may potentially originate from outside of the annotated repeat instances can significantly reduce the number of reads that can be utilized. For example, a single occurrence of a full repeat outside of the annotated instances would lead to exclusion of most consensus sequence reads from the analysis of that repeat type. The RepeatMasker software [25], utilized here for annotating the repeat instances, will normally recognize a full copy of a repeat, preventing such an extreme scenario. The sequences outside of the annotated instance set, therefore, tend to be fragmented portions of the repeats that pass below RepeatMasker thresholds.

To assess the impact of un-annotated repeat instances, we have implemented a masking procedure that excludes reads that can be mapped outside of the annotated repeat instances (see Materials and methods). For the majority of the repeat types, such masking reduces the number of mapped reads by less than 10%; however, in several cases over 80% of the reads are masked (Figure S2 in Additional file 1). The exclusion of such reads, however, has very little impact on the overall enrichment estimates, with less than 1% of the repeat types showing statistically significant differences (Table S3 in Additional file 1).

The ability to distinguish the reads originating from repeat types with a high degree of sequence identity relies on stringent selection criteria that eliminate ambiguously mapped reads. The alterations of the read sequences due to the presence of sequencing errors or SNPs may, therefore, lead to some erroneous read associations. To characterize the frequency of such mis-associations, we have assessed false positive mapping rates, simulating SNP and sequencing error incorporation based on empirically observed frequencies (see Materials and methods). We find that for 99% of the repeat types, the false positive rate due to SNP effects is below 0.029%, and 2.8% for the sequencing errors (Figure S3 in Additional file 1). While the contribution of such errors to the enrichment estimates depends on the individual dataset, the low false positive rates suggest that even four- to five-fold enrichment observed at some of the significantly enriched repeats will not result in the substantial enrichment magnitude at other repeat types. Furthermore, the contribution of sequencing errors, estimated here based on the Illumina 1G error profile, is expected to be lower for the current and future sequencing platforms.

### Phylogenetic analysis of enrichment

In general, a read lacking unique alignment can only be utilized for estimating average enrichment for a set of sequences that contain all potential alignments of that read. The approach described in the previous section employs sets of repetitive sequences classified by the RepeatMasker software as belonging to one of the types annotated in the Repbase database [26]. While such classification groups repeats by sequence similarity, some repeat types often share common sequence and may be hard to distinguish based on the reads available in the dataset. Combining similar repeat types into a single group typically allows utilization of additional reads, providing more precise enrichment estimates at the expense of repeat type granularity.

**Figure 2:**
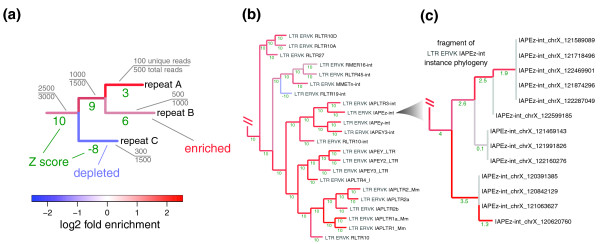
**Phylogenetic analysis of repeat enrichment patterns**. **(a) **Aiming to provide most informative estimates of repeat enrichment that can be attained for a given dataset, repeats are organized into a phylogenetic tree on the basis of read set similarity to maximize the number of uniquely assignable reads that can be used for enrichment estimation. The estimates are illustrated on the resulting tree branches using colors. The nodes of the tree represent sets of repetitive sequences. The gray labels show the fraction of total number of ChIP reads that map to a given set of sequences (node) that can be associated uniquely. The tree is constructed in a way that maximizes the number of additional uniquely associated reads gained at each step. For instance, considering repeats A and B together allows 1,000 uniquely associated ChIP reads to be to utilized for enrichment estimation, even though the sum of the reads uniquely associated with repeat A and repeat B separately is 600. The 400 additional reads are those that map to both A and B repeats, but do not map to any other repeats (in the same way the discarded read in Figure 1 maps to both C and D). The length of each branch corresponds to the number of the unique reads gained using a log scale when collapsing sequences of the descendant nodes into a single set. The statistical significance of the observed enrichment or depletion is shown as a Z-score (green numbers). Large positive Z-score values denote statistically significant enrichment (Z-score of 3.1 corresponds to a *P*-value of 10^-3^), and negative values correspond to significant depletion. The Z-score magnitude is capped at 10. **(b) **A fragment of the enrichment phylogeny of the Repbase repeat types for H3K9me3 enrichment in mES cells. The example illustrates grouping of repeats from ERV-K class, all of which, with the exception of RLTR19-int, are highly enriched for the H3K9me3 modification. Additional examples are shown in Figure S4 of Additional file 1. **(c) **A small fragment of H3K9me3 enrichment phylogeny for the individual instances of the intracisternal A particle (IAP) interspersed repeats (IAPEz-int). The fragment clusters instances located within a specific region on chromosome X due to a high degree of sequence identity between them. While the lack of discriminating sequences precludes evaluation of each instance individually, considering nearly identical instances together allows the demonstration of statistically significant enrichment of this localized group of instances or the H3K9me3 mark in mES cells. LTR, long terminal repeat.

To address a general problem of estimating read enrichment for repetitive sequences, we propose a phylogenetic approach in which enrichment is evaluated using a hierarchy most informative of a particular dataset. An optimal phylogeny would at each step partition a set of sequences in a way that maximizes the number of reads with alignments unique to a single partition. Since the computational cost of such a top-down partitioning approach appears to be prohibitive, we have instead implemented a greedy algorithm, constructing phylogeny from the bottom up by iteratively grouping sequences with the highest read set similarity (see Materials and methods). The results of such analysis are visualized as a tree with branch lengths representing the amount of uniquely aligned reads gained at each step, and colors denoting the enrichment estimates (Figure 2a).

A portion of the repeat enrichment phylogeny constructed for the mES H3K9me3 ChIP data is shown in Figure 2b. The fragment contains repeat types significantly enriched for the H3K9me3 mark, including active intracisternal A particle (IAP) long terminal repeat (LTRs) and early transposons (ETn). The approach progressively groups closely related repeat types based on the gain of reads that can be uniquely associated for the combined groups. For instance, the initial steps group IAP LTRs 1 and 1a (bottom of Figure 2b). When considered individually, a total of 1,991 and 1,758 reads can be uniquely associated with IAP LTR1 and 1a, respectively. Considering these two repeat types together allows a total of 5,664 uniquely associated reads to be used. The extra 1,915 reads gained were previously omitted as they could originate from either IAP LTR1 or 1a (but not any other repeat type, analogous to the omitted C and D common read illustrated in Figure 1). These repeat types are then considered together with IAP LTR2 variants (2, 2a, 2b) to provide an average enrichment estimate for the entire subfamily of repeats. Examples of fragments illustrating enrichment phylogenies of other well-known LTR subfamilies and short interspersed nuclear element (SINE) repeats are shown in Figure S4 of Additional file 1. The high Z-score values and the notable branch lengths of the leaf nodes illustrate that the H3K9me3 dataset can distinguish these Repbase types sufficiently well to provide informative enrichment estimates for each individual type. For instance, the analysis shows that the RLTR19-int repeats (an ERV-K class interspersed LTR) are significantly depleted of the H3K9me3 mark, even though they share reads with the highly enriched set of interspersed ETn repeats.

The phylogenetic methods can be used to analyze enrichment among very similar sequences, such as multiple integrated copies of the same type of transposable element. A section of the mES H3K9me3 enrichment phylogeny for interspersed instances of the IAP repeat IAPEZ-int is shown in Figure 2c. The cluster groups particularly similar instances located on chromosome X. The high degree of sequence identity does not allow evaluation of the enrichment of each individual instance; however, statistically significant enrichment of H3K9me3 can be demonstrated for small groups of these nearly identical chromosome X repeat instances. Examining instance enrichment phylogenies for a number of additional ERV repeat types, we find that such clusters are typical for repeat types with high average enrichment (IAPEy, IAPLTR3, GLN). Complete enrichment phylogenies for these and other repeat types can be browsed on the authors' website [27].

### Repeat enrichment across mouse cell lines

**Figure 3:**
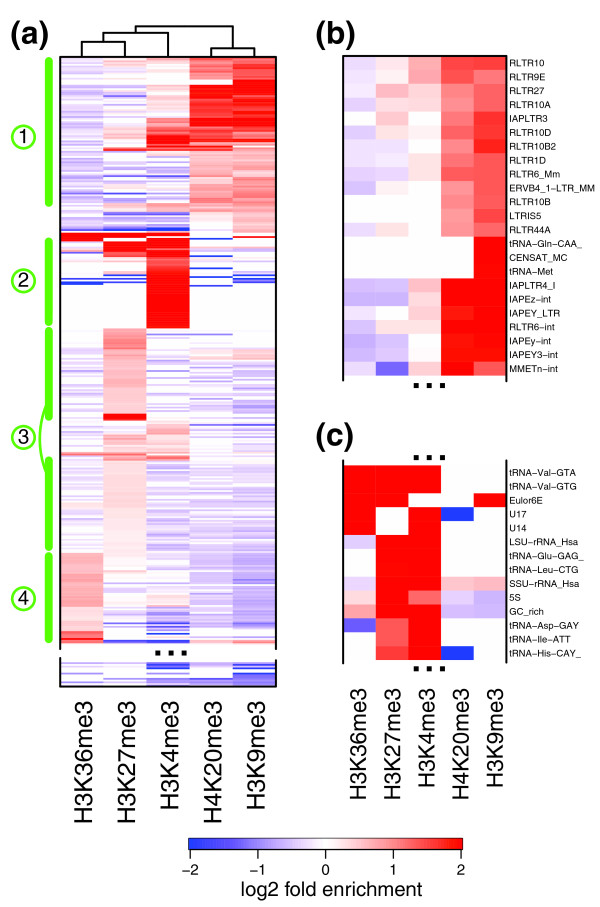
**Repeat enrichment patterns in mouse cell lines**. **(a) **Combinatorial patterns of repeat enrichment in mES cells. The repeat types (rows) were clustered according to the MLE enrichment in different marks (disregarding depletion; see Materials and methods), with red colors corresponding to enrichment, and blue colors corresponding to depletion. Repeat types that do not show statistically significant enrichment or depletion are shown in white. Prominent sets of repeat types are highlighted on the left-hand side (1 to 4; see text). The bottom part of the plot is omitted as it contains repeats devoid of enrichment in any examined modifications. See Figure S5 in Additional file 1 for a complete, magnified view showing all the repeat type labels. **(b) **An enlarged view of a portion of set 1, illustrating ERV1/ERV-K repeats enriched for H3K9me3 and H4K20me3. **(c) **A portion of set 2 showing enrichment for H3K4me3 and H3K27me3 at tRNA repeats.

To examine the combinatorial patterns of repeat enrichment in mES cells, we have clustered Repbase repeat types by their maximum likelihood enrichment estimates (MLEs) for several histone methylation marks associated with transcriptional activation (H3K4me3, H3K36me3), and repression (H3K27me3, H3K9me3, H4K20me3) [4]. The enrichment estimates were calculated relative to the histone H3 background, using combined canonical and instance-mapped reads, masking reads that originated from outside of the annotated repeat regions. A number of prominent clusters of repeat types with similar patterns of enrichment can be seen from this overview analysis (Figure 3a).

Repeat types from set 1 are marked by increased average methylation in H3K9me3 and H4K20me3 marks (Figure 3b). The set is composed almost completely (93%) of the endogenous retroviral repeats. The majority (66%) of these repeats belong to the ERV-K family, which is significantly over-represented within the set (corrected hypergeometric *P*-value < 1e-10). These include IAP LTRs, a particularly active family of retrovirus-like transposable elements that is expressed in early mouse embryos [28]. This pattern of methylation of IAP repeats is in agreement with the original analysis of the data [4] and other experimental assessments [23]. It should be noted, however, that earlier work in mES cells [22] did not observe H3K9me3 enrichment of IAPs. Set 1 also includes early transposon (ETn/MusD) repeats, mouse mammary tumor virus LTRs, and a number of unclassified or putative ERV-K repeat types.

In addition to the ERV-K family, set 1 also contains key members of the ERV1 family, including MuRRS (murine retroviral-related sequences), MuRV-Y (murine repeated virus on Y chromosome), MuLV (type C murine leukemia virus) and LTR-IS (insertion elements) repeat types. It does not, however, include members of the MER4 subfamily of ERV1 (medium reiterated repeat family 4), which are devoid of both H3K9me3 and H4K20me3 enrichment. To ensure that the H4K20me3 enrichment estimates cannot be attributed to utilization of H3 background, we have verified that the observed patterns are maintained when normalized using whole-cell extract data from a mouse embryonic fibroblast (MEF) cell line (Figure S6 in Additional file 1).

The members of the third ERV class (ERV-L) are notably absent from set 1. Instead, these repeat types are over-represented within set 3, which is marked by enrichment in tri-methylation of H3 Lys27 (H3K27me3). Both ERV-L and ERVL-MaLR LTR families are over-represented within the set (*P*-values of 0.04 and 7e-6, respectively). Set 4, a cluster of repeats marked by the tri-methylation of H3 Lys36 (H3K36me3), a mark typically associated with transcriptional elongation, is composed primarily of the SINE repeats (*P*-value < 1e-10), including nearly all types of Alu, B2 and ID subfamilies.

The analysis shows a number of expected enrichment patterns. As observed by earlier studies, the major and minor satellite repeats are enriched for H3K9me3 [4,22,23,29,30]. The majority of the tRNA repeat types show strong enrichment in the H3K4me3 mark associated with active transcription (set 2, Figure 3c). Some tRNA repeats are also enriched for H3K27me3 or H3K36me3. Since the provided methods evaluate repeat enrichment by pooling signal from all repeat instances of that type, the enrichment for both marks does not necessarily indicate that they are present at the same genomic position. Similarly, enrichment for H3K4me3 and H3K9me3 observed for some of the LTR repeats in set 1 (Figure 3a) is unlikely to be found simultaneously on the same physical loci. The most abundant family of repeats, long interspersed nuclear elements (LINEs), does not show a uniform pattern of enrichment, with only HAL1-3A repeats occurring within H3K27me3-enriched set 3.

**Figure 4:**
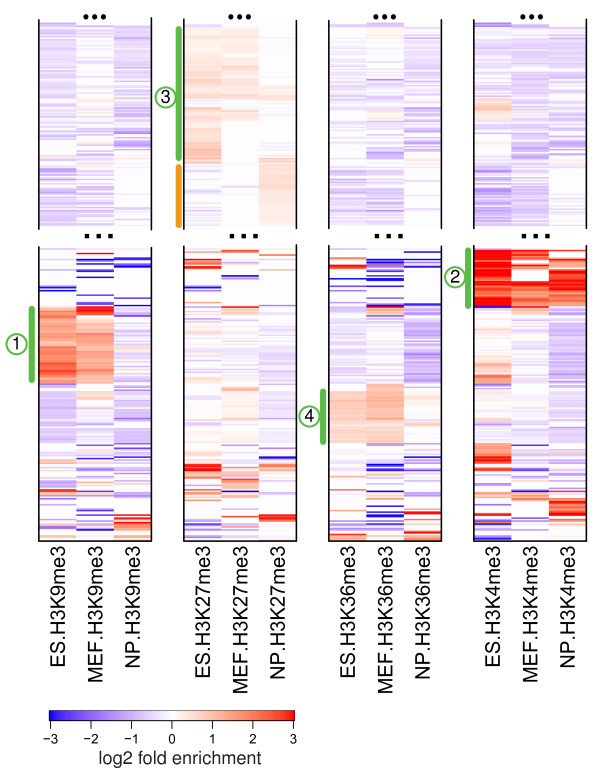
**Comparison of histone modification profiles in mES, neuronal progenitor and mouse embryonic fibroblast cells**. The repeat types were clustered based on their enrichment profiles across four different histone marks in three cells, so that the order of repeat types is the same for each histone methylation mark shown. Green bars mark major clusters of enrichment, with numbers corresponding to mES clusters from Figure 3a. The orange bar marks a set of repeats, composed predominantly of LTRs, that acquires H3K27me3 in NP cells. See Figure S7 in Additional file 1 for a complete plot.

Next, we have compared patterns of histone modifications observed in the mES cells with those found in MEF and neural progenitor (NP) cells (Figure 4). The three cell types show distinct patterns of repeat enrichment, with the differences being particularly striking in the case of the NP cells. The large degree of variation of histone methylation levels between different mouse cell lines has been shown in earlier studies [22], and the widespread reduction of H3K9me3 levels across major repeat families has been noted in the original analysis of these data [4].

We find that most repeat types from mES set 1 maintain statistically significant H3K9me3 enrichment in MEF cells, but show clear absence of H3K9me3 modification in NP cells. Among all examined repeat types, only telomeric and major (gamma) satellite repeats exhibit significant levels of H3K9me3 enrichment across all three cell types. Similarly, the H3K36me3 mark is maintained at most SINE repeat types from set 4 in MEF cells but is lost in NP cells. NP cells, however, do maintain significant H3K4me3 enrichment in many tRNA repeats (set 2). Most repeats from set 3 maintain H3K27me3 enrichment in MEF cells, with only a small fraction of those repeats showing significant H3K27me3 enrichment in NP cells. An additional set of repeat types that acquires H3K27me3 enrichment in NP cells (Figure 4, orange bar) is composed primarily of LTRs (75%). As in the case of H3K27me3-enriched repeat types in mES cells, this new set shows significant over-representation of ERV-L repeats (*P*-value < 1e-4).

The mES enrichment estimates utilize H3 background, whereas MEF and NP estimates are normalized using whole-cell extract data. To ensure that the chromatin changes described above cannot be attributed to the normalization differences, we have repeated the analysis normalizing mES ChIP data using whole-cell extract data from MEF cells, and find that the choice of background does not affect the observed chromatin state differences (Figure S8 in Additional file 1).

### Repeat enrichment patterns in human CD4+ T cells

**Figure 5:**
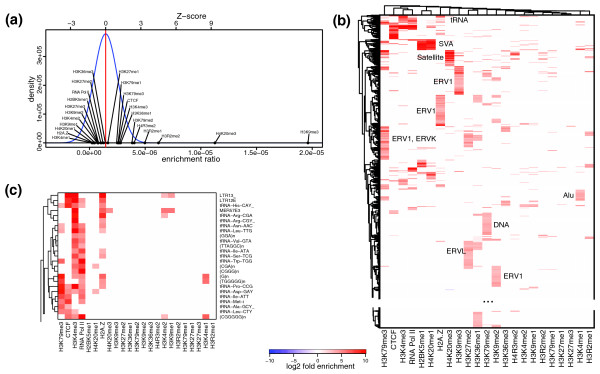
**Repeat enrichment patterns in human CD4+ T cells**. **(a) **To normalize read counts in the absence of input sequencing data, the enrichment values were estimated relative to other marks using distributions of enrichment coefficients observed for each repeat type (see Materials and methods). The plot shows enrichment coefficients for various chromatin marks for the HSAT6 repeat. H3K9me3 and H4K20me3 deviate from the nominal levels exhibited by most of the marks in a statistically significant way. **(b) **Similar to Figure 3a, the repeat types were clustered according to their combined enrichment estimates across measured chromatin marks. Part of the plot containing no enrichment clusters is omitted (see Figure S9 in Additional file 1 for a complete plot). Repeat families over-abundant within major clusters are labeled on the plot. **(c) **Enlarged view of the tRNA-dominated cluster located at the top of the plot shown in (b).

To examine the patterns of repeat-associated chromatin profiles in human cells, we have utilized an extensive set of histone methylation marks measured by Barski *et al. *in CD4+ T cells [3]. The dataset interrogates 20 lysine and arginine methylations of histone tails, the H2A.Z histone variant, RNA polymerase II and CTCF binding sites. It does not, however, provide an input control measurement, which is imperative for normalization of the signal associated with the repetitive elements. We therefore estimated enrichment values using repeat-type specific distributions of scaled read counts (see Materials and methods; Figure 5a). Such calculations are based on the assumption that, for a given repeat type, most of the measured chromatin marks will not exhibit statistically significant enrichment levels, and will instead provide a normalized base-level that would ordinarily be assessed using the input measurements. While this method cannot be used to establish statistically significant depletion, it allows identification of repeat types that exhibit significant enrichment in some of the examined marks.

The combinatorial patterns of resulting repeat enrichment estimates are shown in Figure 5b. As in the analysis of the mouse data, we find that repeats belonging to the same family frequently exhibit a common pattern of histone modifications. In this way, 92% of Alu repeat types belong to a cluster distinguished by statistically significant enrichment in H3K4me1, in contrast to earlier observations of widespread enrichment for H3K9me2 in Alu repeats [31]. All of the SVA repeats show enrichment in H4K20me1 and H2BK5me1. As expected, all five types of satellite repeats are enriched for H4K20me3, with HSAT6 (Figure 5a) and HSAT III and I also enriched for H3K9me3 [32]. Similarly, the snRNAs appear within the clusters marked by RNA polymerase II binding, and the majority of tRNA repeats show enrichment in H3K4me3 (Figure 5c).

The repeats from the ERV1 family comprise most of the types in disjoint clusters marked by H3K9me3, H3K9me2 and H2A.Z enrichment. ERV1 repeats are also over-represented (*P*-value = 8e-4) within a set defined by H3K79me3, which also contains more than half of all ERV-K repeat types (*P*-value = 8e-8). In contrast, the repeats types belonging to the ERV-L family show a distinct enrichment in H3K27me2 modification.

## Discussion

The substantial amount of sequence divergence found both between and within specific repeat types, combined with knowledge of the genomic integration context of many repeat instances, allows estimation of enrichment patterns unique to individual repeat types, small sets of highly homologous repetitive elements, and in some cases individual repeat instances. The developed phylogenetic method provides a way to examine enrichment at multiple levels of such a hierarchy, starting with small sets of repetitive elements grouped according to their similarity in the context of the current data. The method uses a greedy algorithm in an attempt to derive a phylogenetic tree that provides the most informative enrichment estimates. Such a heuristic approach does not guarantee optimality; however, direct optimization carries prohibitive computational costs. Alternative phylogenetic trees can also be derived - for example, based on input reads or different distance metrics. We find, however, that the utilized distance measure provides a straightforward assessment of how similar two sequence sets appear in terms of the reads being analyzed.

For the standard classification of repetitive sequences defined by Repbase types, we show that the inclusion of reads associated with known repeat instances can lead to an order-of-magnitude increase in the number of usable reads, allowing identification of statistically significant enrichment for many more additional repeat types. Despite independently derived enrichment estimates, our analysis of repeat enrichment in multiple histone marks illustrates that, within both mouse and human cells, there are specific chromatin states associated with larger repeat families.

Some of these patterns, such as common enrichment of different tRNA repeat types for marks associated with active transcription, are expected given the functional roles of these loci. Other signatures may be attributed to the genomic context. For example, H3K4me1 enrichment common to human Alu repeats is likely to reflect the strong bias of Alu repeats to reside within GC-rich, gene-dense regions where such methylation is typically found [3,12,33]. Chromatin signatures shared by different types of interspersed transposable elements may also be a consequence of silencing mechanisms targeting these repeat families. This is likely to be the case for the histone methylation patterns observed for the ERV families.

The analysis of mouse data illustrates two distinct histone modification patterns by ERV repeats. While the repeats belonging to ERV-K and ERV1 subfamilies appear within the cluster enriched for H3K9me3 and H4K20me3 modifications [4,23,34], repeats from the ERV-L and ERVL-MaLR families are instead enriched for H3K27me3. Both patterns are characteristic of repressive chromatin state but are established by two different mechanisms: one associated with constitutive heterochromatin [35,36] and the other with Polycomb-mediated silencing of euchromatic domains [37]. Studies of mES mutants deficient for the Suv39 h histone methyltransferase have noted that decreased H3K9me3 levels in the repetitive elements were accompanied by a marked increase in H3K27me3 enrichment, suggesting potential functional compensation of the two mechanisms [21].

The difference between silencing mechanisms is consistent with earlier studies of IAP ERV-K and ERV-L retrotransposons. They showed that even though both types of repeats are actively transcribed following global DNA demethylation at the two-cell stage, the ERV-L transcripts and IAP silencing do not occur at the same stage [28]. The ERV-L transcripts decline significantly by the eight-cell stage whereas IAP silencing is only apparent at the blastocyst stage. It is tempting to speculate that ERV-L silencing would not be tied to DNA re-methylation [23]; however, DNA methylation has been found for both ERV-L and ERV-K repeats [38,39].

The disparity between ERV-L and the other two classes of ERVs can also be seen in the analysis of the human data, where ERV-L repeats show enrichment in H3K27me2, while ERV1 and ERV-K repeat types are marked by di- and tri-methylation of H3K9, H2A.Z and H3K79me3. Such similarity is surprising given the significant differences in transposition rates, the presence of active transposable elements between the two organisms, as well as the amount of variation seen among different types of mouse cells [12,13].

Many of the identified chromatin enrichment patterns recapitulate results of earlier studies (see Table S4 in Additional file 1 for details). This includes well-established enrichment for heterochromatic marks in satellite repeats [4,22,23,29,30], or enrichment of H3K9me3 in ERV1/ERVK classes of repeats [4,23,34]. Similarly, our comparison of the histone modification patterns of repetitive elements between three mouse cell types confirms widespread differences noted previously [4], which are particularly striking in the case of NP cells. An earlier study by Martens *et al. *[22] examined histone methylation levels of select repetitive elements in mES and MEF cells, and mES cells transformed with retinoic acid. While their results also indicate extensive variation between cell types, there is notable disagreement in the estimated levels of enrichment. These include the absence of H3K9me3 and H4K20me3 in the IAP repeats of MEF cells, and an overall lack of concordance of these two modifications across examined repeat types and cell lines.

While the exact mechanisms involved in silencing of diverse repeat families remain to be elucidated, it is clear that chromatin state plays an important role in suppressing activity of ERVs and other transposable elements. The presented methods allow isolation of the signal associated with specific sets of repetitive elements, which enables analysis of chromatin state and its variation across different repeat types. Further evaluation of epigenetic marks associated with the repetitive elements will be important, especially given potential involvement of transposable elements in disease [18,40]. To facilitate such analyses, the methods proposed in this work were implemented as a web application, allowing for estimation of enrichment of annotated repeat types and visualization of enrichment phylogenies. In addition to analysis of ChIP-seq data, the developed methods can be used in the context of other types of high-throughput sequencing experiments, most notably for analysis of copy-number variation of repetitive sequences from comparative genomic sequencing data [41].

## Materials and methods

### Associating reads with annotated repeat types

A read was associated with a particular repeat type if it satisfied the following criteria: 1, the read aligned to a single or multiple locations within the canonical sequence of that repeat type, or annotated instances of that repeat type within the genome, incorporating 13 bp (half of read length) genomic sequences flanking the annotated instances; 2, no alignment of such or better quality to canonical or instance sequences associated with any other annotated repeat types could be obtained. An optional masking procedure, designed to exclude reads potentially originating from un-annotated repeat types, added a third requirement: 3, no alignment of such or better quality to any portion of the genome assembly that is not associated with the annotated instances of that repeat type could be obtained.

The set of annotated repeat types was taken from the Repbase database [26]. The repeat instances were determined based on the RepeatMasker scan (default parameters, as provided at [25]). The procedure was implemented using modified SeqMap [42] and bwa [43] aligners, in combination with custom repeat assembly files. The combined repeat assembly file contained a single FASTA entry for each repeat type as defined in the Repbase database. The sequence of each entry is composed of the canonical repeat sequence concatenated with all instance sequences identified by the default RepeatMasker scan, separated by spacer blocks of 80 'N' characters. Similarly, the canonical repeat assembly contained only canonical repeat sequences, and the instance-only repeat assembly contained only sequences identified by the RepeatMasker. The aligner (SeqMap and bwa) implementation was modified to record the number of reads that map, possibly more than once, only to a single FASTA entry corresponding to a specific repeat type. When utilizing masking option (condition 3), reads that could be aligned to the regions outside of the RepeatMasker-detected instances were excluded from the analysis. The mm9 assembly was used for mouse data, the hg18 assembly for human data. In all analyses, only alignments with at most one mismatch were admitted.

### Dataset size estimation

The effective size of the dataset was estimated as the number of reads that map at least once to the genome assembly or canonical repeat sequences.

### Estimation of enrichment coefficients

The 95% confidence interval (CI) of the fold-enrichment ratio was based on a Poisson model with non-informative Bayesian prior [44]. Specifically, the MLE was determined as:

θ^=C(s+12)S(c+12)

where *s *is the number of signal (ChIP) reads mapping to a given repeat type, *c *is the number of control reads, *S *is the size of the signal dataset, and *C *is the size of the control dataset. The confidence interval bounds were calculated as [λ(1−α2),λ(α2)], where:

λ(x)=θ^⋅F[x,2(s+12),2(c+12)]

*F *being the F-distribution, and *α *= 0.05. The estimation of enrichment in mES cells was done relative to the histone H3 data; whole-cell extract data was used for MEF and NPC cell types.

### Comparison of enrichment estimates

Enrichment estimates were compared as a ratio of two binomial proportions, adjusted for the dataset size. The ratio confidence intervals were calculated using a non-iterative approximate Bayesian method proposed by Price and Bonett [45].

### Visualization

To explore combinatorial patterns of enrichment (Figures 3, 4 and 5), the repeat types were clustered according to the MLE estimates. Enrichment values of repeats that do not show statistically significant enrichment (based on 95% CI) were set to 0. The clustering was determined using the Ward method [46], using only positive enrichment values (treating all negative values corresponding to depletion as 0).

### Estimation of mis-alignment rates

The sequencing error rates were estimated from the H3K9me3 ChIP data, based on the mismatches observed when aligning the data to the reference genome assembly. The error frequencies were estimated for each position of the 32-bp reads (ranging from 5.4e-3 in the first position to 1.0e-2 in the last), and mismatch frequencies were estimated for each pair of nucleotides. To estimate the false positive and false negative rates of alignment in the repeat analysis, the repeat assembly was sampled using the error model described above, with 100-fold coverage. Analogous 100-fold sampling was performed to analyze the effect of SNPs, using an SNP frequency of 8.3e-5 (chromosome 1, 5-Mbp average observed SNP rate [47]).

### Relative normalization for human data

As the utilized human CD4+ T-cell ChIP-seq experiments from Barski *et al. *[3] did not include an input measurement, we have estimated enrichment of each repeat type based on the assumption that most of the 21 measured marks should not be enriched in each given repeat. Enrichment estimates were determined by first calculating the percentage of ChIP reads aligned to a repeat type relative to the side of the signal data set. Then, for each repeat type, the percentage ratios calculated for the 21 chromatin marks were used to create a Gaussian distribution. To reduce the effect of the outliers, five marks with the highest percentage ratios were omitted in the estimation of the Guassian.

The distributions did not show significant outliers with low percentage ratios. This is expected since the reduction of the read ratios associated with significant depletion would be necessarily smaller in magnitude than the background percentage ratio for a given repeat, and is likely to be smaller than the variation of the background percentage ratio between different ChIP measurements due to the presence of significant enrichment elsewhere in the genome. Furthermore, none of the examined marks are known to show high genome-wide levels of enrichment, and are therefore not expected to show a high magnitude of depletion.

To account for variation stemming from small read counts, each percentage ratio was sampled 100 times based on the Beta distribution. The parameters of the Gaussian distribution were then determined using all percentage ratios, and used to calculate enrichment Z-scores for each chromatin mark.

### Phylogenetic analysis of enrichment

The phylogenetic trees (Figure 2) were constructed using bottom-up clustering, starting with the leaves. Each node of the tree corresponds to a set of repetitive sequences. The trees represent a hierarchical grouping of repetitive elements into successively larger sets. The leaves represent initial sets of sequences: Repbase repeat types in the case of Figure 2b; individual instances of MMERGLN repeats in the case of Figure 2c. The enrichment estimates for each set are calculated based on the reads that can be uniquely associated with that set (see conditions 1 and 2 above). In deriving phylogenetic trees for a set of repeat instances (Figure 2c), reads mapping to repeat sequences outside of the considered instance set were discarded.

The trees were constructed using the neighbor-joining method. The distance between nodes (sets of sequences) was determined as a ratio of reads shared by the two sets to the total number of reads aligning to both sets. The trees shown here were calculated using ChIP data. For a given node, the branch length was calculated as a number of uniquely assignable reads in a given node that were not unique in the descendant nodes. The branches were colored according to the enrichment MLE.

### Availability

To facilitate analysis of enrichment patterns in repetitive regions by other groups, we have implemented the developed methods in a web application. The implementation allows processing of simple ChIP-control paired or more extensive datasets for a variety of organisms. The application can be accessed on the authors' website [48].

## Abbreviations

bp: base pair; ChIP: chromatin immunoprecipitation; CI: confidence interval; ERV: endogenous retrovirus; ETn: early transposon; IAP: intracisternal A particle; LTR: long terminal repeat; MEF: mouse embryonic fibroblast; mES: mouse embryonic stem cell; MLE: maximum likelihood estimate; NP: neural progenitor; SINE: short interspersed nuclear element; SNP: single nucleotide polymorphism.

## Authors' contributions

PVK designed the study; PVK and DSD implemented the algorithms; LJL developed the web applications; PVK wrote the manuscript with the help of PJP and DSD.

## Supplementary Material

Additional file 1 **Supplementary figures and tables**. A combined set of supplementary figures and tables referenced in the manuscript. Click here for file
