# Supplementary material for: Estimating enrichment of repetitive elements from high-throughput sequence data
Source: Genome Biol. 2010 Jun 28;11(6):R69. doi: 10.1186/gb-2010-11-6-r69 (PMC2911117; doi:10.1186/gb-2010-11-6-r69)

Supplementary Tables

Supplementary Table 1: Agreement between canonical and instance enrichment estimates for different statistical stringency thresholds.
The table shows fraction of repeat types for which confidence intervals of enrichment estimates based on canonical and instance sequences overlap (i.e. do not disagree).

	H3K27me3	H3K36me3	H3K4me3	H3K9me3	H4K20me3
**75% CI**	67.44	76.05	67.33	74.58	73.63
**90% CI**	84.87	93.28	82.35	90.13	90.23
**95% CI**	88.87	95.80	85.71	94.33	94.64
**99% CI**	93.91	98.32	91.07	96.85	97.06
**99.9% CI**	96.22	99.26	94.64	98.32	98.32

Supplementary Table 2: Canonical and instance enrichment estimates: agreement of significant enrichment calls.
For the repeat types significantly enriched based on the instance-sequence estimates, the table shows the fraction of these repeat types that are also significantly enriched according to the canonical-sequence estimate (first number in each cell). The second number shows the reverse comparison: the fraction of repeat types significantly enriched according to the canonical sequence estimates that are also enriched according to the instance-sequence estimates.

	75% CI	90% CI	95% CI	99% CI	99.9% CI
**H3K27me3**	52.8 / 68.9	46.7 / 72.4	42.8 / 74.8	38.0 / 77.3	35.2 / 74.1
**H3K36me3**	32.3 / 55.1	24.1 / 62.5	23.8 / 72.0	22.5 / 83.3	19.5 / 90.0
**H3K4me3**	75.0 / 69.9	61.5 / 72.7	57.4 / 69.2	51.7 / 67.6	39.0 / 55.5
**H3K9me3**	66.6 / 71.8	69.3 / 86.9	68.3 / 87.8	70.5 / 89.7	67.72 / 92.4
**H4K20me3**	64.0 / 66.6	70.8 / 84.2	72.5 / 90.4	70.7 / 93.0	68.0 / 88.3

Supplementary Table 3: Agreement of enrichment estimates with and without masking of reads potentially originating outside of the annotated repeat instances. For mES cell data, the table shows agreement between enrichment estimates obtained with and without masking of reads that may originate outside of the un-annotated repeat instances.

measure	H3K27me3	H3K36me3	H3K4me3	H3K9me3	H4K20me3
Fraction of repeat types with intersecting CIs	0.996	0.997	0.991	0.994	0.995
Fraction of repeat types significantly enriched according to the un-maksed estimates that are also significantly enriched based on masked estimates	0.982	0.955	0.964	1.00	1.00
Fraction of repeat types significantly enriched according to the maksed estimates that are also significantly enriched based on un-masked estimates	0.979	0.955	0.989	0.963	0.952

Supplementary Table 4: Agreement with previously published results.
The table summarizes agreement of enrichment observations for specific mouse repeats with earlier studies.

observations	studies in agreement	studies in disagreement
H3K9me2 in major and minor satellites	[29] [30] [23] [22] [4]	
H3K9me3 in ERV1/ERV-K LTRs	[4]	
in IAP (ERV-K) LTRs	[4] [23]	[22] (agrees on H4K20me3 enrichment)
in ETs/MusD LTRs	[4] [23] [34]	
H3K27me3 in ERV-L repeats		
H3K4me3 in tRNA repeats	expected	
lack of H3K9me3 and H4K20me3 in LINE, SINE repeats	[22] [4]	
H3K36me3 in SINE repeats	expected	

(note: citation numbers are taken from the References section of the main manuscript)

Supplementary Figures

Supp. Figure 1. Agreement between canonical and instance enrichment estimates.
The plot shows fraction of repeat types for which canonical and instance-based enrichment estimates are in agreement (i.e. not significantly different), for various statistical stringency thresholds (x-axis).

Supp. Figure 2. Exclusion of reads potentially originating from outside of the annotated repeat regions.
The plots show the fraction of reads masked in analysis of ES ChIP data and WCE data in MEF and NP cells. Each plots shows a cumulative distribution function, with the % of masked reads given on the x-axis, and the fraction of repeat types for which the number of masked reads was below that value on y-axis. For the vast majority of the repeat types, under 10% of reads are masked. However there are some repeat types for which 60% or even 80% of reads are masked.

Supp. Figure 3. Estimates of alignment error rates due to SNPs and sequencing errors.
The figures show cumulative distributions of false positive (a,c) and false negative (b,d) rates of read mis-alignment due to presence of sequencing errors (a,b), or SNPs (c,d). The figure d. omits 5 outlier repeats with high false negative rates. The inset of figure d. shows the cumulative distribution including these outliers.

Supp. Figure 4. Additional examples of phylogenetic enrichment tree fragments.
The plots illustrate additional examples of repeat type enrichment phylogeny fragments, such as that shown in Figure 2b of the main manuscript. **a.** A fragment of H3K9me3 enrichment tree, grouping several prominent LTR repeat types with close exogenous relatives, including murine leukemia virus (MuLV), virus-like 30S retroelements (MMVL30), as well as murine Y-chromosome associated retrovirus (MURVY). **b.** A small fragment of the H3K9me3 enrichment phylogeny, grouping LTR-IS subtypes. The Gypsy subfamily repeat LTR81A has only 64 uniquely associated reads, sharing additional 27 reads with the LTRIS2-5 repeats. Despite the large fraction of shared reads, LTR81A does not exhibit enrichment for H3K9me3 observed in LTRIS repeats. **c.** A fragment of the H3K36me3 enrichment phylogeny illustrating a clustering of SINE B1 repeats showing statistically significant enrichment for this transcriptional elongation-linked mark. The fragment also includes a subtree containing several highly similar LTR ERVs (RLTR25A, B, *etc.*) which altogether are not enriched for the mark.

Supp. Figure 5. Repeat enrichment estimates in mES cells.
The plot shows a full-scale version of Figure 3a of the main manuscript.

Supp. Figure 6. Repeat enrichment in mES cells using MEF WCE background.
The cluster plots below compare the repeat enrichment patterns obtained using H3 backround (**a**, as in Figure 3a of the main text), and using whole-cell extract (WCE) from the MEF cell line (**b**.). The results illustrate that the difference in the

H4K20me3 enrichment patterns is minimal and does not impact the observations presented in the manuscript.

Supp. Figure 7. Repeat enrichment across mouse cell types.
The plot shows a full-scale version of Figure 4 of the main manuscript.

Supp. Figure 8. Repeat chromatin states in different cell mouse cell lines, comparing mES estimated using mES H3 and MEF WCE backgrounds.
The cluster plots below compare the repeat enrichment patterns across three mouse cell lines (Figure 4 of the main text), illustrating the impact of using MEF WCE background to normalize the mES data. **a.** Analysis using mES H3 backround (as in Figure 4 of the main text) for mES estimates. **b.** Analysis using MEF WCE background for mES calculations. The NP and MEF enrichment estimates are obtained using their corresponding WCE datasets, in both a. and b. The results illustrate that the observed differences are not specifically attributed to use of H3 background in mES enrichment estimates.

Supp. Figure 9. Repeat enrichment in human CD4+ T-cells.
The plot shows a full-scale version of Figure 5 of the main manuscript.

Supp. Figure 1.

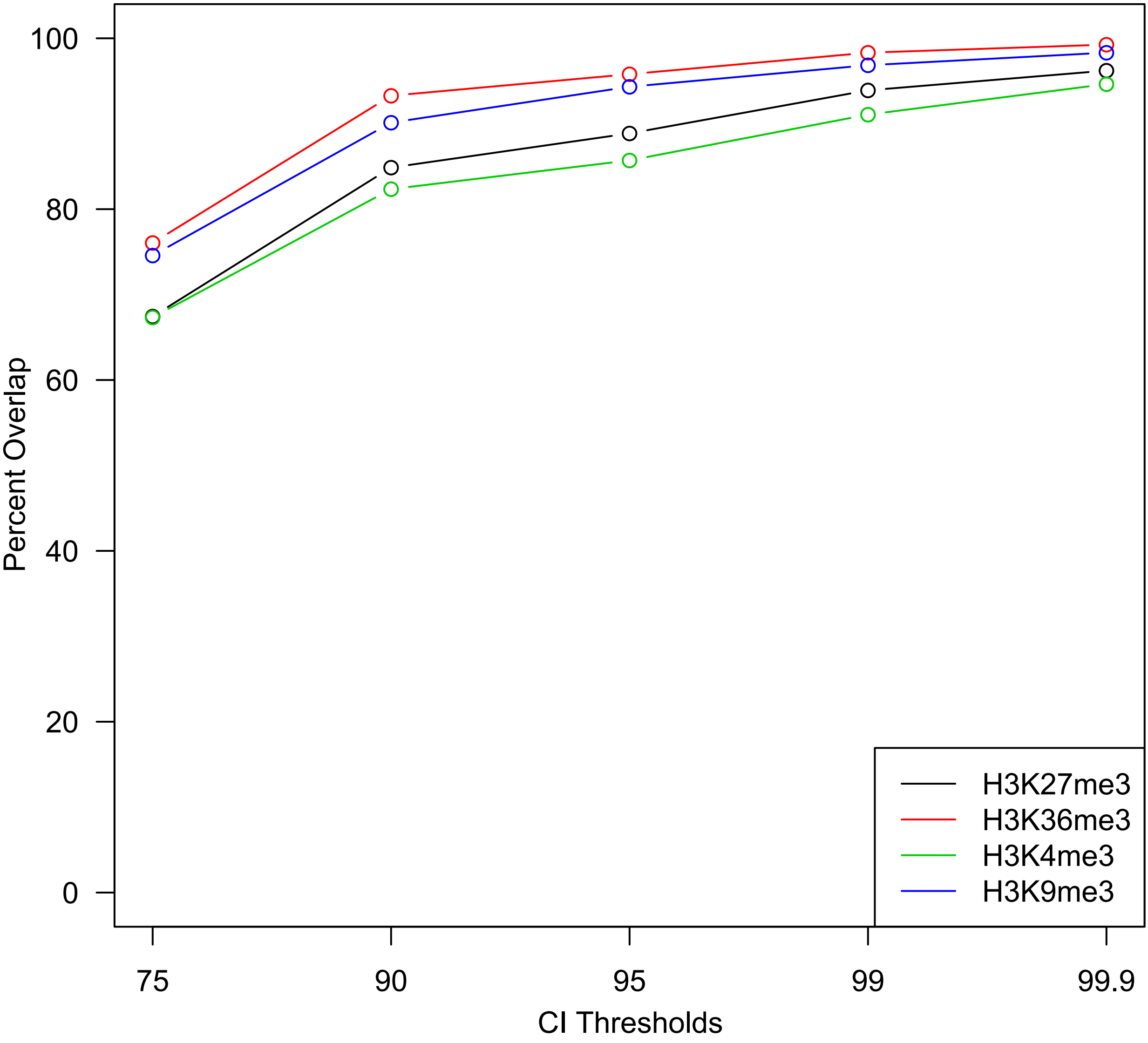

Supp. Figure 2.

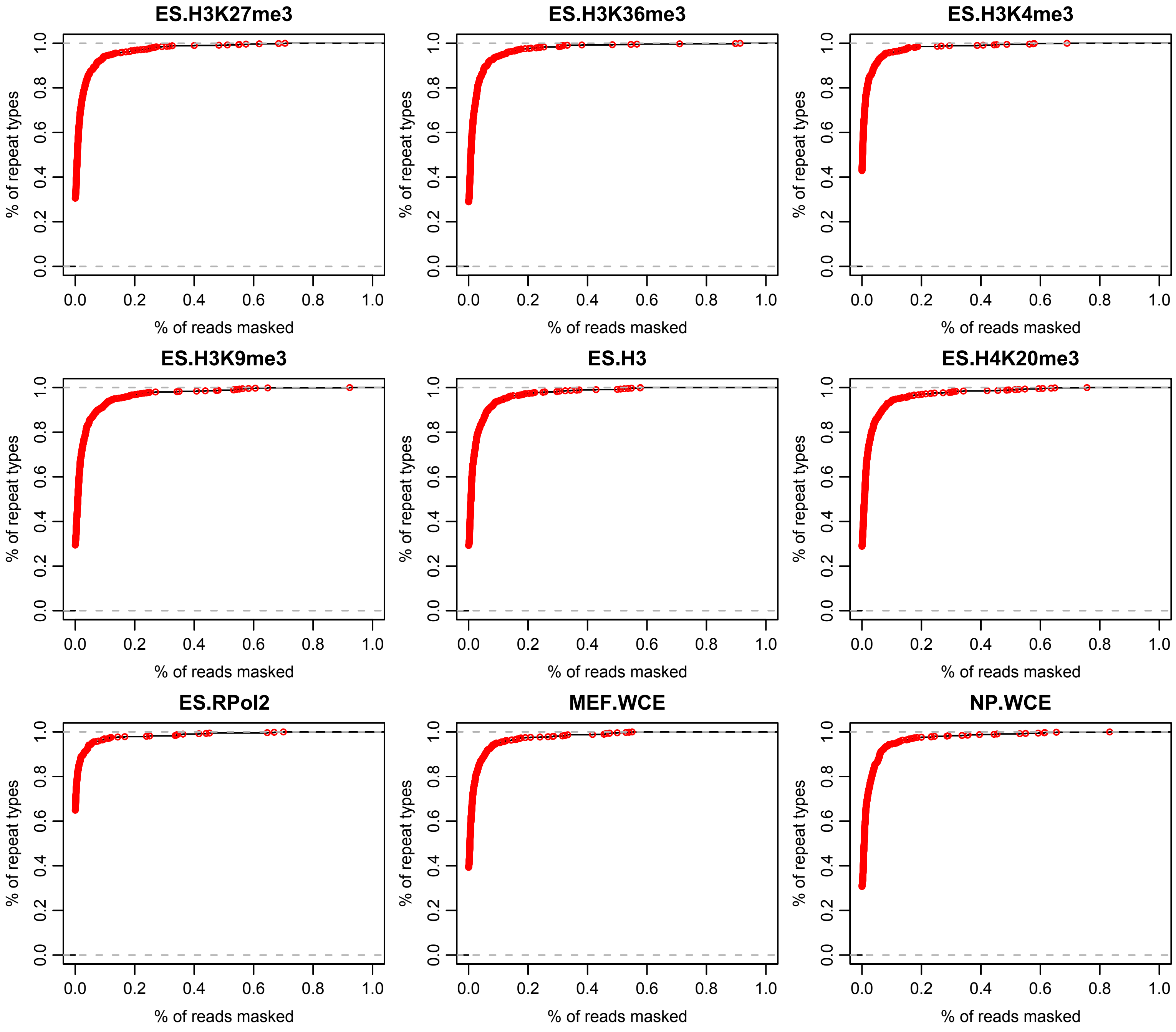

Supp. Figure 3.

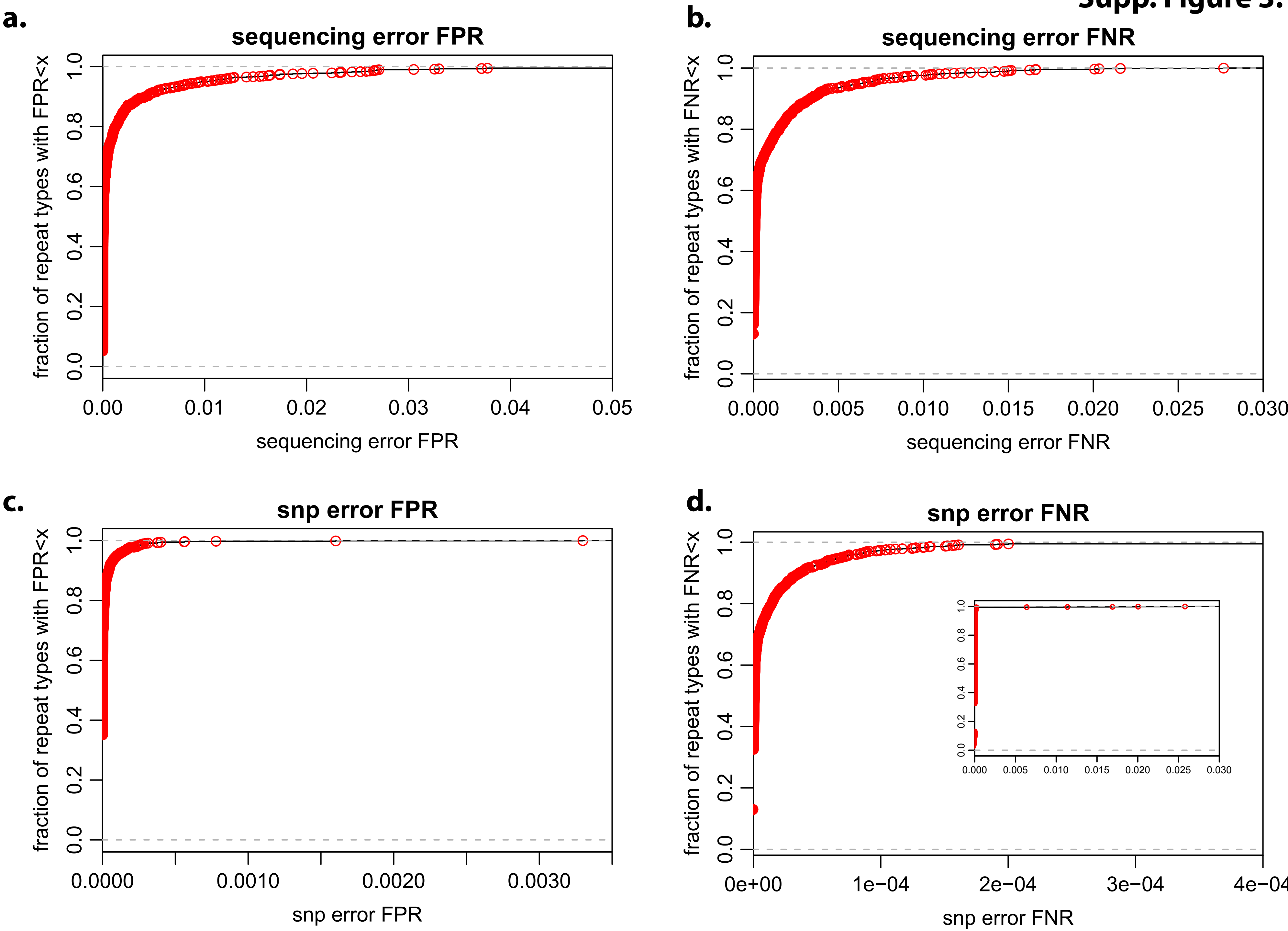

Supp. Figure 4.

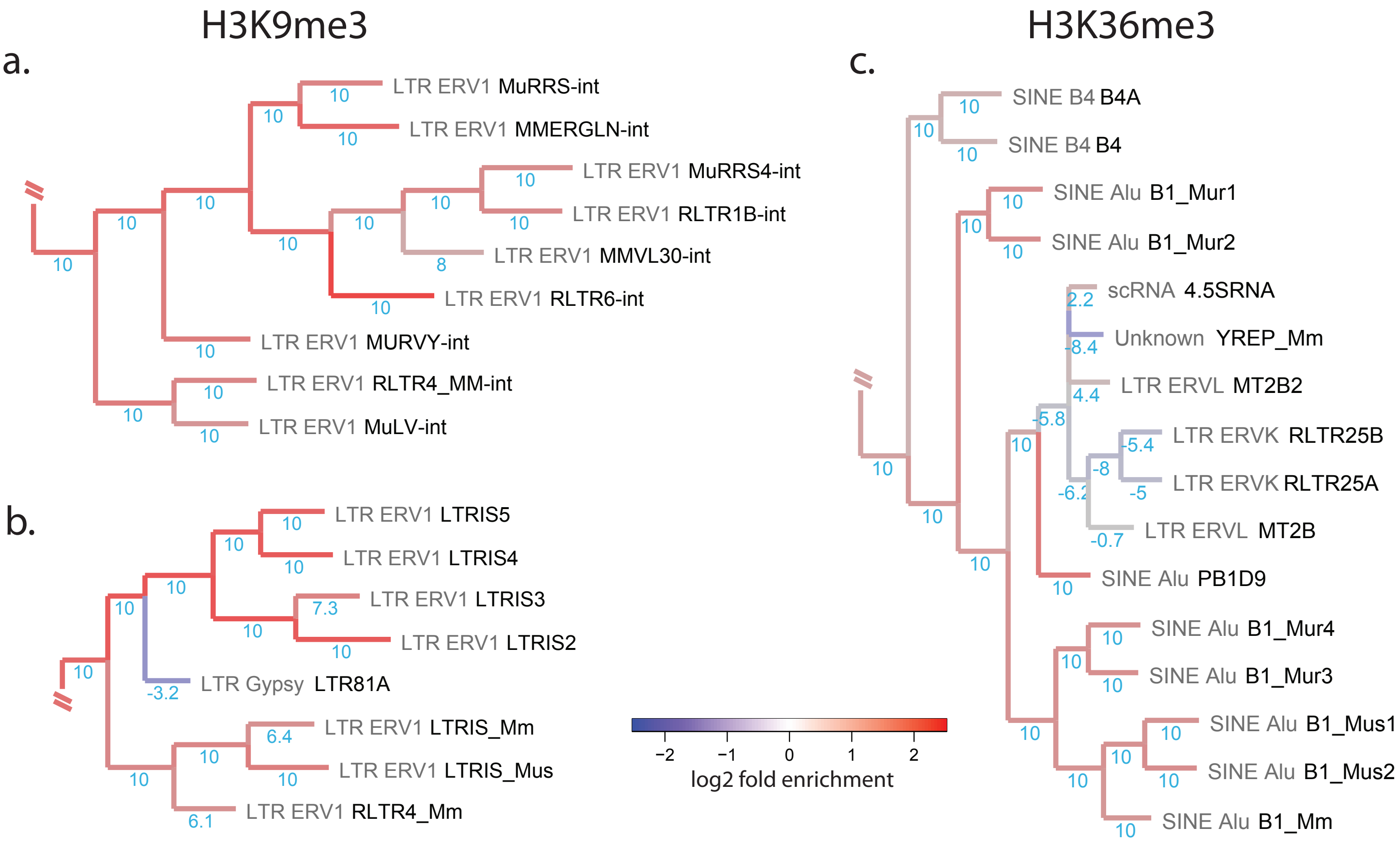

Supp. Figure 5.

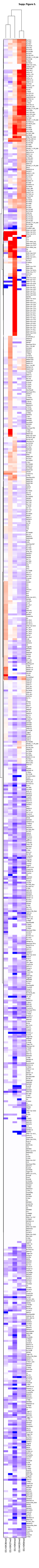

Supp. Figure 6.

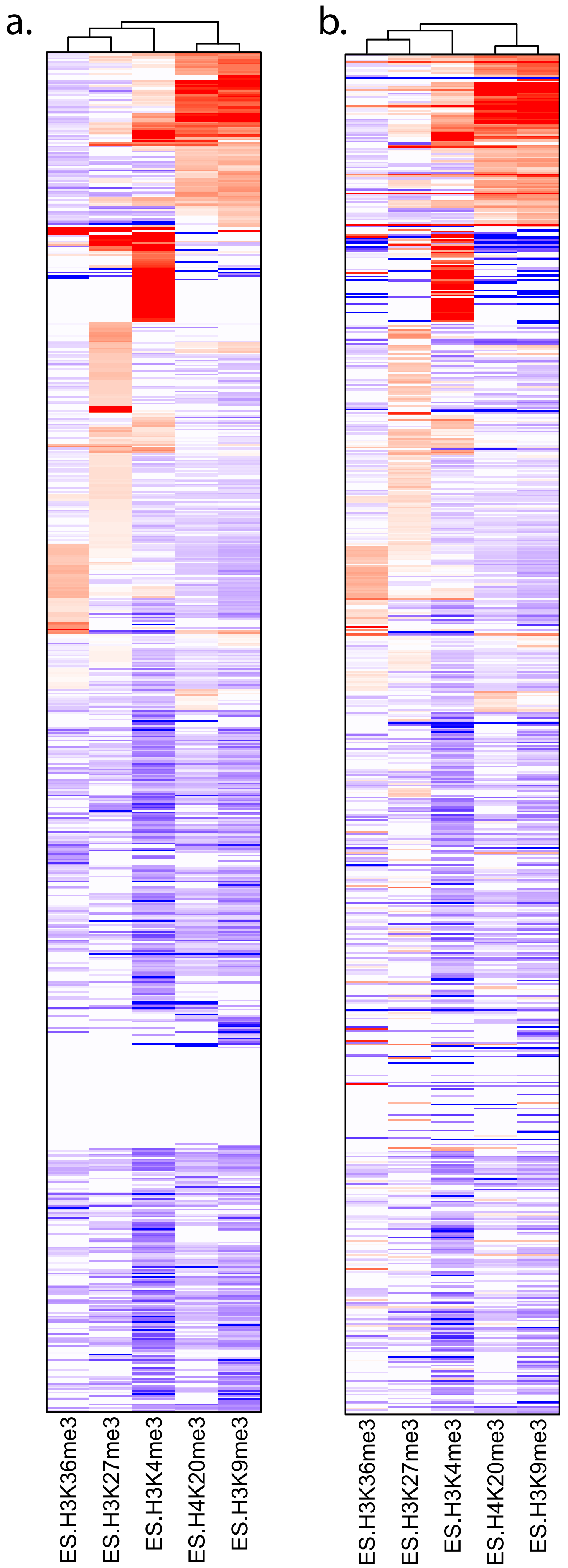

Supp Figure 7.

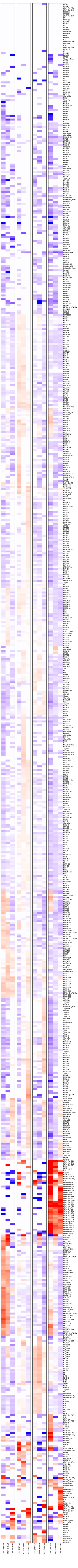

Supp. Figure 8.

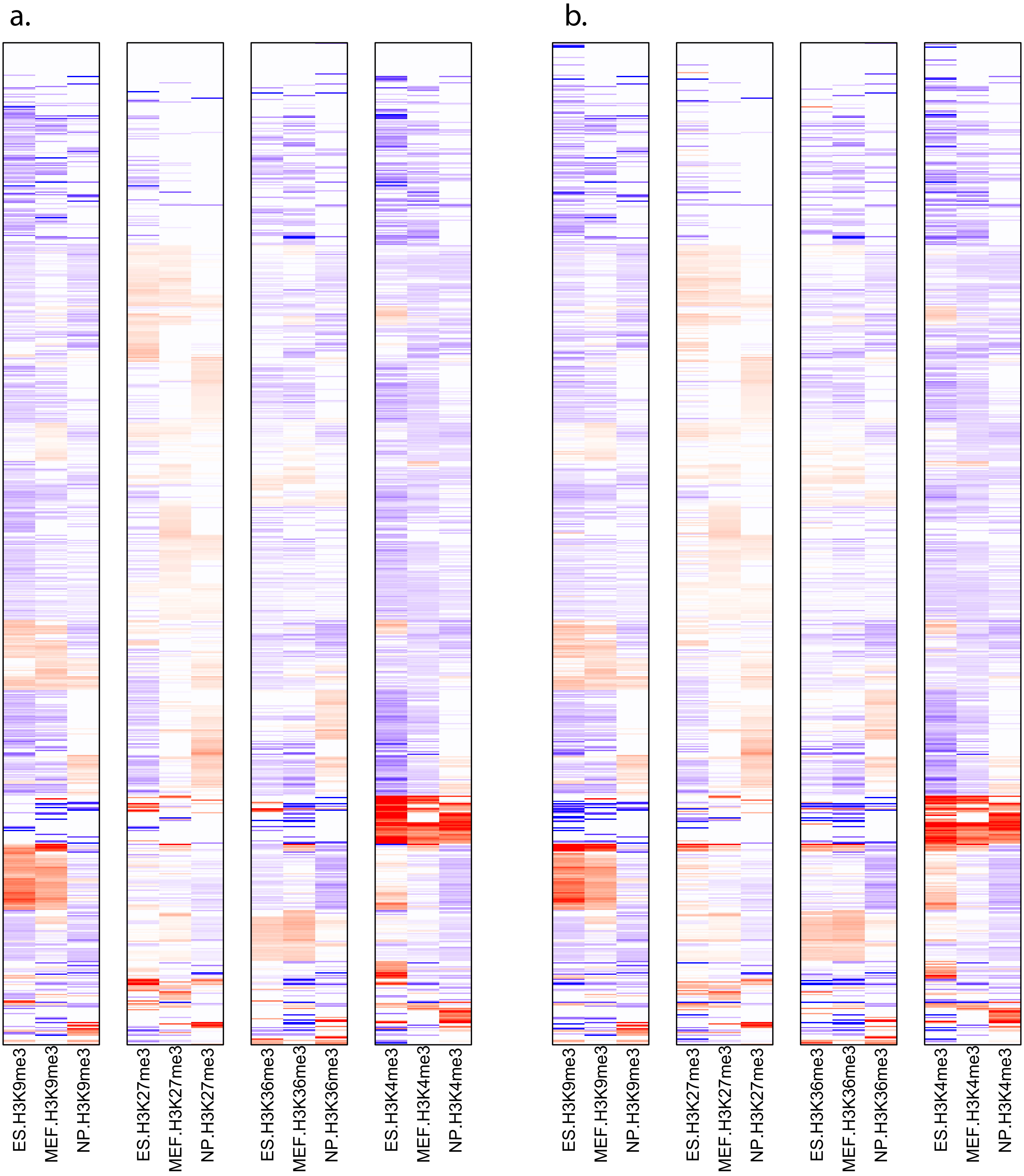

Supp. Figure 9.

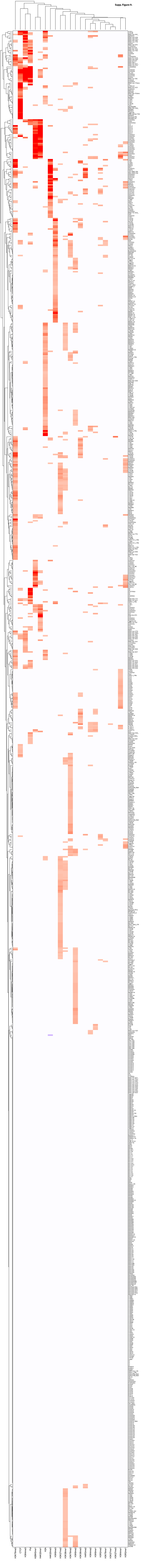

Supplement: Additional file 1 — Supplementary figures and tables. A combined set of supplementary figures and tables referenced in the manuscript. [file gb-2010-11-6-r69-S1.PDF]
